# Synthetic lethals in HIV: ways to avoid drug resistance: Running title: Preventing HIV resistance

**DOI:** 10.1186/s13062-015-0044-y

**Published:** 2015-04-17

**Authors:** Michel Petitjean, Anne Badel, Reiner A Veitia, Anne Vanet

**Affiliations:** Univ Paris Diderot, Sorbonne Paris Cité, F-75013 Paris, France; CNRS, UMR7592, Institut Jacques Monod, F-75013 Paris, France; MTI, INSERM UMR-S 973, F-75013 Paris, France; Atelier de Bio Informatique, F-75005 Paris, France

**Keywords:** Synthetic lethals, Drug targets, Drug design, RNA viruses

## Abstract

**Background:**

RNA viruses rapidly accumulate genetic variation, which can give rise to synthetic lethal (SL) and deleterious (SD) mutations. Synthetic lethal mutations (non-lethal when alone but lethal when combined in one genome) have been studied to develop cancer therapies. This principle can also be used against fast-evolving RNA-viruses. Indeed, targeting protein sites involved in SD + SL interactions with a drug would render any mutation of such sites, lethal.

**Results:**

Here, we set up a strategy to detect intragenic pairs of SL and SD at the surface of the protein to predict less escapable drug target sites. For this, we detected SD + SL, studying HIV protease (PR) and reverse transcriptase (RT) sequence alignments from two groups of VIH^+^ individuals: treated with drugs (T) or not (NT). Using a series of statistical approaches, we were able to propose *bona fide* SD + SL couples. When focusing on spatially close co-variant SD + SL couples at the surface of the protein, we found 5 SD + SL groups (2 in the protease and 3 in the reverse transcriptase), which could be good candidates to form pockets to accommodate potential drugs.

**Conclusions:**

Thus, designing drugs targeting these specific SD + SL groups would not allow the virus to mutate any residue involved in such groups without losing an essential function. Moreover, we also show that the selection pressure induced by the treatment leads to the appearance of new mutations, which change the mutational landscape of the protein. This drives the existence of differential SD + SL couples between the drug-treated and non-treated groups. Thus, new anti-viral drugs should be designed differently to target such groups.

**Reviewers:**

This article was reviewed by Neil Greenspan Csaba Pal and István Simon.

**Electronic supplementary material:**

The online version of this article (doi:10.1186/s13062-015-0044-y) contains supplementary material, which is available to authorized users.

## Background

Classical SL genetic interactions involve non-lethal mutations (carried by two or more genes) whose combination leads to cell death. They have been extensively used to study gene-product interactions in the secretion pathway of yeast [1] and bacteria [2]. Then, they were used to develop anti-cancer therapies [3-6] by pinpointing a gene (say, X) whose inactivation forms a pair of SL with a mutated cancer-causing gene. In this context, the drug will target gene X and not the gene responsible for the disease. The synthetic lethality relationship appears when the product of gene X is rendered non-functional by the action of the drug. Thus, the existence of both non-functional proteins provokes a lethal phenotype and leads to cancer cell death. The effect of the drug on normal cells, would not change their phenotype, and thus should not induce any secondary effects. Based on this paradigm, we describe a slightly different concept to uncover new druggable targets in RNA viruses using an intragenic SL-based strategy [7]. Indeed, RNA-viruses can escape drugs [8] and vaccines [9], due to mutation of the targets against which such therapeutic molecules are developed. To circumvent this problem, pocket-binding drugs targeting viral fundamental functions should be pinpointed, so that the virus cannot mutate without losing the relevant essential function (Figure four in [6]). Invariant residues fulfill this condition but they are rare in the proteins of RNA-viruses. It is this notion, of “invariance”, that we extend to a group of residues. Intragenic SL and synthetic deleterious (SD) can be exploited for this purpose. For simplicity, we call SDL the ensemble of SD + SL. A group of amino acids, spatially close (say, less than 10 Å between two residues) and located at the protein surface, can provide a suitable therapeutic target. These residues should be either invariant or being members of the same SL group. Due to these two features, essentiality for protein function and invariance, these targets are unique in that they might minimize or even prevent viral escape to treatment.

Various studies have been performed to describe pair-wise and higher-order site correlations within RNA-virus proteins [10-17] employing various approaches such as information theory, non synonymous versus synonymous mutations, Bayesian networks, etc. Using generalized kernel ridge regression and maximum entropy models, others [17,18] have described a general and interesting concept, which is the fitness landscape. Unfortunately, their goal was not to make the difference between compensatory mutations (CM) and SL pairs. Moreover, they were not interested in pointing to potential druggable sites, which is one of our main aims here. Further works were specifically developed on the viral RNA SL but they simulate them rather than detecting them [19,20].

In a preliminary work taking the HIV protease as a model, [7,21,22], we described positions involved in SDL couples. The method used yielded results comparable to those obtained by other teams working on the same subject [23-25]. However, the sole knowledge of the amino acid (AA) positions is just part of the molecular picture and knowing the exact nature of the AAs involved in the SDL couples is just as important. Moreover, a SDL couple is not expected to exist alone, but rather within the context of a mutational network involving other couples of SDL and CM. Finally, to uncover functional covariation we must exclude background linkage disequilibrium (BLD). In sum, from a sequence alignment and a three-dimensional structure, we developed a strategy involving statistical tests, phylogeny, 3D structure and binding sites for constructing an *in silico* tool that predicts potential therapeutic targets. This tool has been tested on two HIV proteins, the protease (PR) and the reverse transcriptase (RT) and allowed us to describe five targets consisting of SL and invariant positions that should greatly minimize the emergence of drug resistance.

## Results and discussion

### Steps to predict drug targets in silico

**Figure 1:**
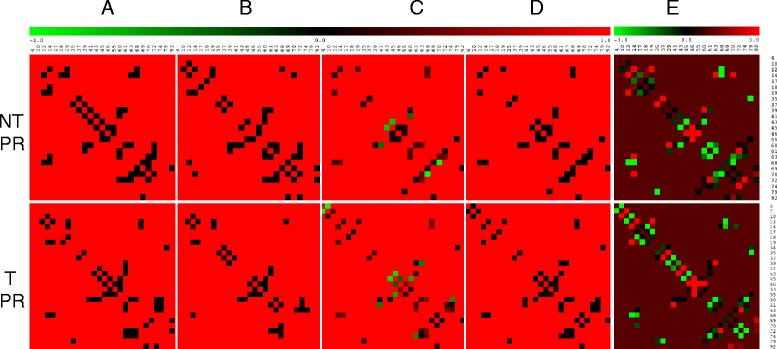
**Accessible covariation studies.** Variant and accessible positions were tested by pair for covariation, if both positions are separated by less than 10 Å. 4 different statistic tests were performed for both sets (sequence from treated T, and non-treated NT patients) and both proteins (PR: protease, RT: reverse transcriptase): **A**: Fisher exact test, p-values <0.05 are shown. **B**: ${\chi}_{ij}^2$ p-values <0.05 are shown. **C**: D’, first described by Lewontin [39] and used to detect gametic disequilibrium. This result is given if Θ is >1.5 or <0.5 [14]. The D’ coefficient has a value between −1 and 1. **D**: the correlated coefficient r^2^. The result is given if Θ is >1.5 or <0.5 [14]. The r^2^-correlated coefficient has a value between 0 and 1. **E**: D’ coefficient was calculated for non-synonymous pair of positions (A-A) as well as synonymous pair of positions (S-S). Their ratio, D’_AA/SS_ identifies the background linkage disequilibrium or in other words the fact that the sequences share a common ancestor. The result is given positive if D’_AA/SS_ > 2.

To define protein regions as potential druggable targets avoiding therapeutic escape, we have focused on SDL couples (Figure four in [6]) and invariant positions located in their vicinities. To do so, seven steps are necessary. We need first to identify pairs of interdependent sites. They were defined by examining the variant positions (those having accumulated more than 0.3% of mutations). Specifically, these variant positions were tested in pairs using statistical tests, described in the Material and Methods (MM) section, commonly used to define dependencies between positions. Couples responding positively to 3 of the 4 tests were taken as interdependent pairs. A couple of residues may co/anti-vary for two main reasons: they can be either an interdependent couple (CM or SDL) or be derived from BLD. Only SDLs qualify for druggable targets not allowing therapeutic escape. SDL couples were defined as those having a number of observed pairs of mutated residues smaller than the number of expected pairs. Thus, we have defined a dissimilarity coefficient ξ, which is negative for SDL couples, and positive for CM couples (see Material and Methods). We filtered the results of this exploration to keep only the pairs located on the protein surface as it is the most accessible location for known therapeutic targets (accessibility threshold greater than 25%, using the ASA software [26] and implemented by Allan *et al.* [27] based on the 3D PR structure PDB ID:1HSG [28] and 3D RT structure PDB ID:1DLO [29]). Next, we had to prove that SDL couples did not derive from a common ancestor (i.e. exclude BLD). Position couples underwent a further test: for all codons underlying these two positions, we computed the number of synonymous (S) and non synonymous (A) mutations. If the number of non-synonymous mutation pairs (A-A) was twice as much the number of synonymous mutation pairs (S-S) we considered that this pair of codons undergoes a positive selective pressure. Such pairs were therefore assumed not to derive from a common ancestor, in other words, not derived from BDL (Figure 1E for PR and 2 in brown for RT). Keeping in mind the idea of suggesting druggable targets, we retained only SDL couples that were close in space (at less than 10 Å on the 3D structures). Finally, “invariant” positions (<0.3% of mutations at the relevant position relative to the ancestral sequence), although infrequent, can also be taken into account in the design of inescapable drug targets. Thus, all invariant positions being at less than 10 Å from SDL positions were also kept. The last step was to determine the drugability of a group of residues. As a first approach, we used the Q-SiteFinder software to list most important binding sites of a protein from its 3D structure.

### Predicted drug targets

**Figure 2:**
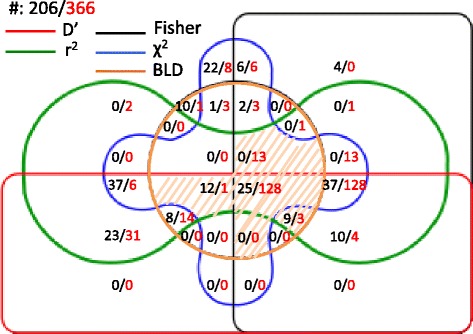
**Venn representation of the interdependent pairs in the RT protein.** All variant pairs of residues close in space and at the surface of the protein were examined using five statistical tests. Black numbers stand for the results from non-treated set. Red numbers represent the results from treated set of patients. χ^2^, D’, r^2^ and Fisher’s are statistical tests explained in MM. BLD is a background linkage disequilibrium described in MM. The shaded area corresponds to the pairs of interdependent residues, having 3 valid statistical tests out of 4 and a BLD score > 2.

**Figure 3:**
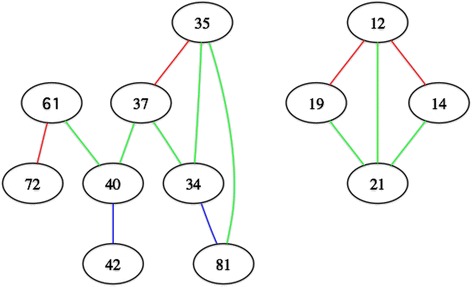
**Graph representation of SDL and invariant interactions in PR-NT.** 2 sub-graphs derived from our computational analysis, composed of 4 and 8 positions. A link between two position means that these two positions are at the surface of the protein and close in space. Moreover, a red link binds two SDL position. A green link binds a SDL position to an invariant position. Finally, a blue link binds two invariants positions. The numbers correspond to the HIV PR positions.

**Figure 4:**
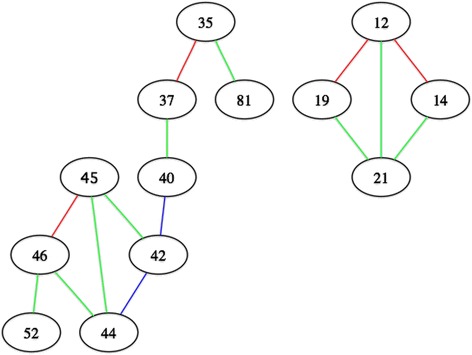
**Graph representation of SDL and invariant interactions in PR-T.** 2 sub-graphs derived from our computational analysis, composed of 4 and 9 positions. A link between two position means that these two positions are at the surface of the protein and close in space. Moreover, a red link binds two SDL position. A green link binds a SDL position to an invariant position. Finally, a blue link binds two invariants positions. The numbers correspond to the HIV PR positions.

**Figure 5:**
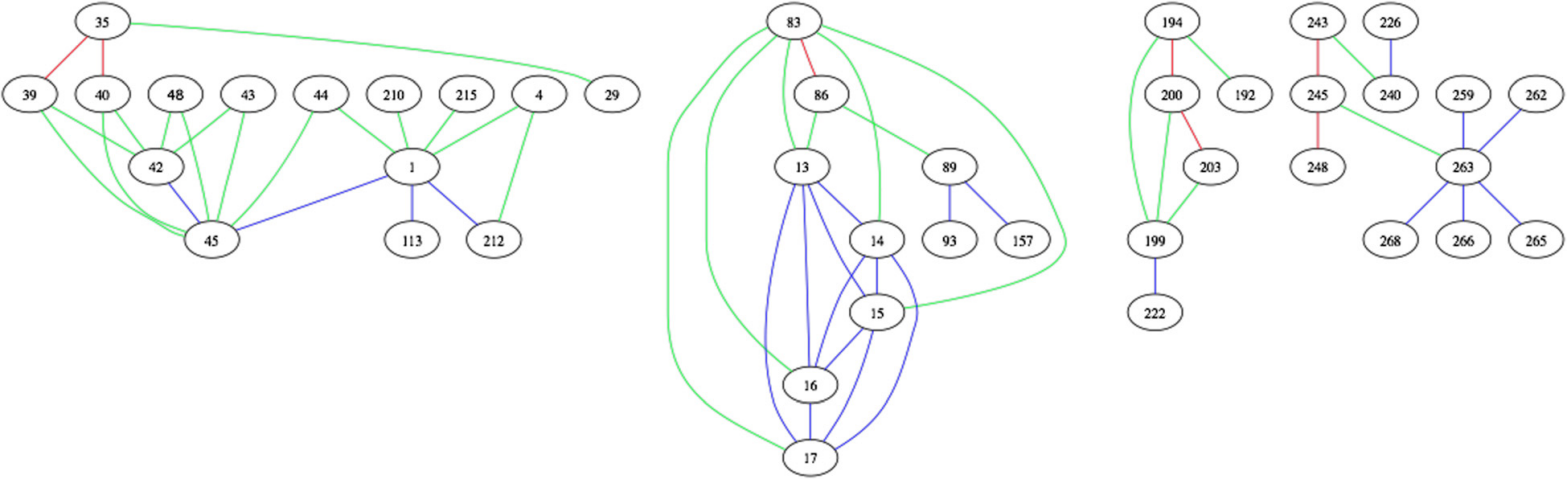
**Graph representation of SDL and invariant interactions in RT-NT.** 4 sub-graphs derived from our computational analysis, composed of 6, 10, 11 and 15 positions. A link between two position means that these two positions are at the surface of the protein and close in space. Moreover, a red link binds two SDL position. A green link binds a SDL position to an invariant position. Finally, a blue link binds two invariants positions. The numbers correspond to the HIV RT positions.

**Figure 6:**
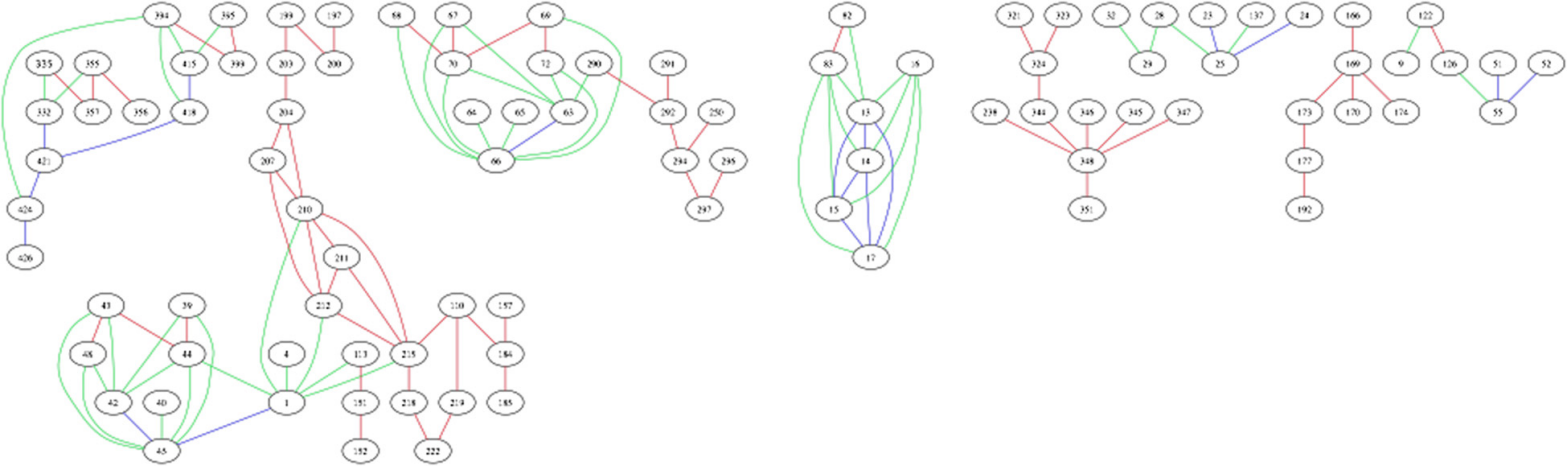
**Graph representation of SDL and invariant interactions in RT-T.** 8 sub-graphs derived from our computational analysis, composed of 6, 7, 7, 7, 10, 13, 16 and 29 positions. A link between two position means that these two positions are at the surface of the protein and close in space. Moreover, a red link binds two SDL position. A green link binds a SDL position to an invariant position. Finally, a blue link binds two invariants positions. The numbers correspond to the HIV RT positions.

From sequence alignments (PR-NT/protease-non treated group: 24656 sequences, PR-T/protease-treated group: 10585 sequences, RT-NT: 23052 sequences and RT-T: 9784 sequences), all pairs of variant positions close in space, on the surface were tested for interdependence. To this end, we used four tests: the Fisher’s exact test (Figure 1A for PR and 2 in the black area for RT), χ2 (Figure 1B for PR and 2 in the blue area for RT) D’ (Figure 1C for PR and 2 in the red area for RT), r^2^ (Figure 1D for PR and 2 in the green area for RT). When a pair of variant positions passes with success 3 of 4 statistical tests, it becomes tagged as interdependent. Figure 1E for PR and Figure 2 in the brown area for RT, represent the BLD, the weaker it is, the bigger is the chance for a pair of interdependent residues to come from a common ancestor. The pairs successful for 3 statistical tests and not derived from a common ancestor are represented on a heatmap for PR (Figure 1) and a Venn Diagram for RT (Figure 2). We compared our results with those of *Rhee et al.* [30]. Of the 49 interdependent pairs they describe for patients under anti-RT treatments, only 5 are close in space and on the surface of a RT. These five couples are positive for our interdependence tests but two of them where rejected by our BLD test. Indeed, this test was not performed by *Rhee et al.* Concerning the PR, out of 49 interdependent couples described by *Rhee et al.* [30] only one is close in space and at the PR surface. We found this positive one with our algorithm. Our previous results [7] and those of three other groups [23-25], were also confirmed by this new strategy, excepted those coming from BLD. Finally, to distinguish between SDL and CM, we determine the dissimilarity coefficient ξ for each pair of residues of each couple (Additional file 1: Table S1 represents this result for PR and Additional file 2: Table S2 for RT). All intermediate results, from the validity of statistical tests to the SDL determination are displayed in Table 1. These results show that half of the interdependent couples come from a common ancestor. For the other half, only 50% involved SDL couples. To identify groups of positions that will become our future targets, the invariant positions located within 10 Å of a SDL couple were determined. The number of SDL is 10 times higher in the RT-T groups than in the other three groups. This result comes from the fact that the RT is 5 times longer than the PR, and because the sequences of the treated groups contain more mutations. Next, SDL couples and the invariant positions in their vicinity were gathered to form a graph. The subgraph positions of these graphs (in Table 2) represent the potential future targets. PR-NT and PR-T graphs (Figure 3 and 4) contain two subgraphs, the RT-NT (Figure 5) graph has three and RT-T (Figure 6) graph, seven. Note that, subgraphs containing only two positions were excluded because they cannot form a realistic binding site.

**Table 1:** **SDL determination process**

	**PR**		**RT**	
	NT	T	NT	T
Number of interdependent couples	19	23	130	296
Number of interdependent couples excluding BLD	7	8	46	145
Number of SDL couples excluding BLD	4	5	7	63

BLD: Background Linkage disequilibrium.

SDL: Synthetic deleterious and lethal.

PR: Protease.

RT: reverse trancriptase.

NT: non treated.

T: treated.

**Table 2:** **SDL + INV groups**

		SDL + Inv*	**Binding site**	**Site Volume**
Protease	NT	35, 61, 37, 72, 40, 34, 42, 81	40 + 42 + 61	103
		12, 14, 19, 21	12 + 14 + 19	82
	T	35, 37, 81, 45, 40, 46, 42, 52, 44		
		12, 14, 19, 21	12 + 14 + 19	
		63, 72		
Reverse Transcriptase	NT	35, 39, 40, 48,43, 44, 210, 215, 4, 29, 42, 1, 45, 113, 212		
		83, 86, 13, 89, 14, 93, 157, 15, 16, 17	13 + 14 + 15 + 86 + 17	243
		194, 200, 192, 203, 199, 222		
		243, 226, 245, 240, 259, 262, 263, 268, 266, 265	259 + 262 + 263 + 266	375
		6, 9		
	T	394, 395,33, 355, 415, 399, 332, 357, 326, 418, 421, 424, 426		
		199, 197, 203, 200, 204, 207, 210, 211, 43, 39, 212, 110, 157, 48, 44, 4, 113, 215, 184, 42, 40, 1, 151, 218, 219, 185, 45, 152, 222		
		68, 67, 69, 70, 72, 290, 291, 64, 65, 63, 292, 250, 66, 294, 296, 297	63 + 64 + 65 + 66 + 67 + 70 + 72	252
		82, 83, 16, 13, 14, 15, 17	13 + 14 + 15 + 17	243
		321, 323, 324, 238, 344, 346, 345, 347, 348, 351		
		32, 28, 23, 137, 24, 29, 25		
		166, 169, 173, 170, 174, 177, 192		
		122, 9, 126, 51, 52, 55		
		226, 228		
		243, 245		
		101, 237		

*at the protein surface and close in space.

### Are these targets really druggable?

**Figure 7:**
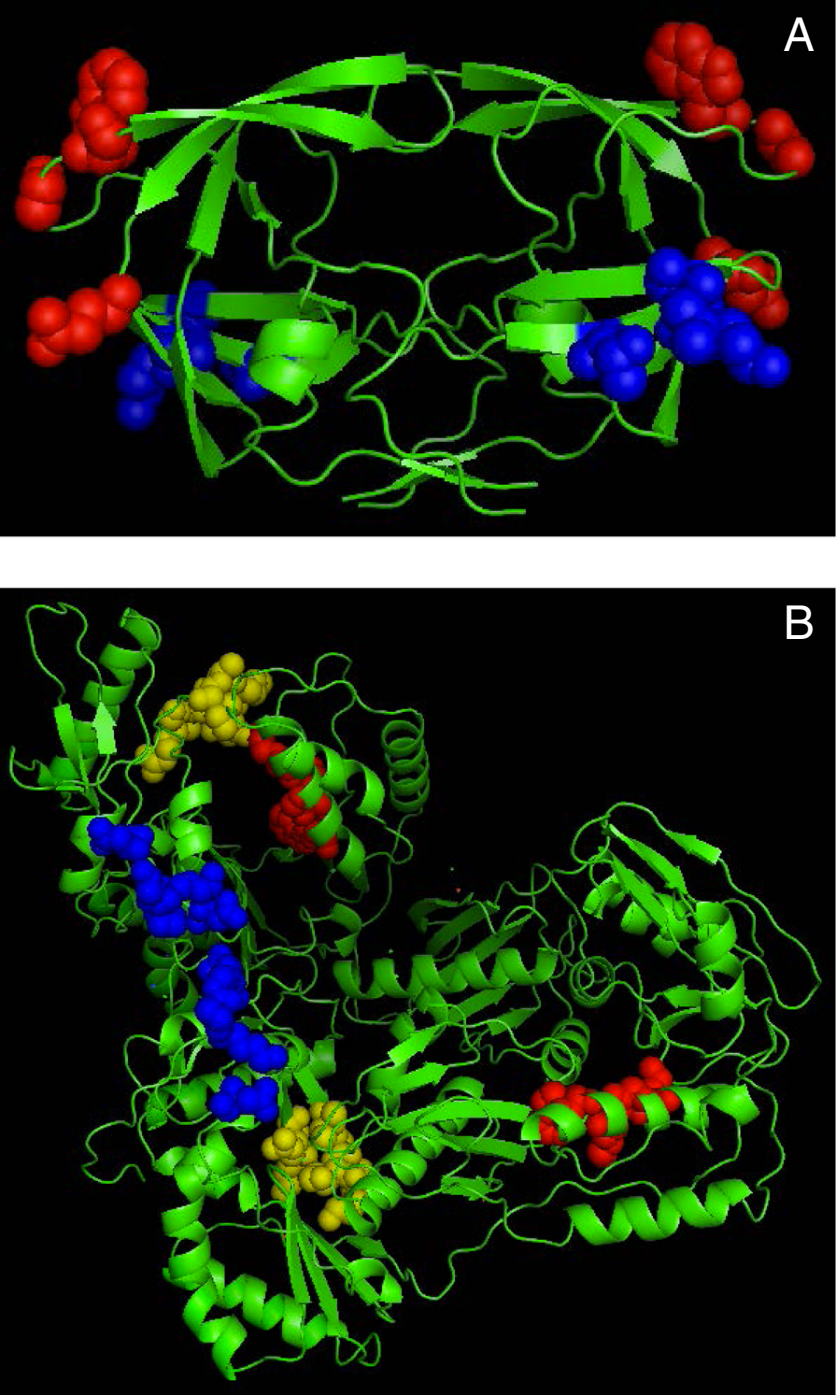
**3D view of the potential target sites. A**: The different target sites are shown on a 3D representation of the HIV-1 homodimer (1HSG) PR. The red target is lining a pocket necessary for the opening of the flaps. The blue one in located in the fulcrum of the protein. **B**: The different target sites are shown on a 3D representation of the HIV-1 homodimer (DLO1) RT. The red and the yellow targets are lining a pocket necessary for docking DNA. The color residues are those constituting of SDL groups and forming a binding site according to the results given by the Q-siteFinder software. The 3D molecules of the A and B panel were built by pymol software [43].

The groups of positions composing these subgraphs are predicted to be good targets to avoid resistance. However, to be of therapeutic interest, these targets should also be a good binding sites, i.e. pocket-shaped and composed of atoms that a small molecule can bind. As a first approximation, we tested this possibility by using the Q-siteFinder program [31]. From a three-dimensional PR structure chosen from the Protein data bank, Q-siteFinder determined 10 protein regions, which could form a binding pocket. We then kept the positions in the intersection between our subgraph results and Q-SiteFinder binding pockets. Table 2 lists the AA groups that fulfill the 7 conditions described at the beginning of this section. These groups therefore are candidate therapeutic targets forming predicted good binding sites with low potential to generate drug-resistance. We have highlighted two of these groups on the PR structure (Figure 7A). The first one, containing positions 12, 14, 19 (T1 in blue on Figure 7A, numbered in Table 2), has a site volume of 103 Å^3^ and is common for patients treated and untreated patients. The second one containing positions 40, 42, 61 (T2 in red on Figure 7A, numbered in Table 2) with a site volume of 82 Å^3^, can only be used for untreated patients. Interestingly, studies of Bonhoeffer’s [17] group on fitness landscape, described the same regions and defined them as characterized by strong epistasis. These regions have previously been described as being important for protein function [32]. The two best-scoring targets defined by the Q-SiteFinder software, correspond to the active site of the PR. The majority of drugs (not to say all) against this protein bind to its active site but, unfortunately, resistance against all these molecules have appeared. Besides, we did not find SDL in those areas. We have highlighted three of these groups on the RT structure (Figure 7B). The first one, containing the positions 13, 14, 15, 86, 17 (T3 in blue on Figure 7B, numbered in Table 2), has a site volume of 243 Å^3^ and is common for treated and untreated patients. Of note, the position 86 disappears from the treated group. This target is localized in the RT fingers. The second one, localized in the thumb and containing positions 259, 262, 263, 266 (T4 in red on Figure 7B, numbered in Table 2) with a site volume of 375 Å^3^, only appears in the untreated set. The last one, involving positions 63, 64, 65, 66, 67, 70, 72 (T5 in yellow on Figure 7B, numbered in Table 2) with a site volume of 252 Å^3^ and localized in the RT palm, is relevant for the treated set only. Interestingly, the second and third targets are involved in the DNA binding process.

Thus far, these results do not tell us anything about the nature of the AAs involved in these couples. Indeed, a given position can be involved in both CM and SDL relationships (concerning two different AA) with other positions and these relationships are interdependent. For this reason, we also compiled a list of the specific AAs involved in all the SDL and the CM couples, because they influence the general mutational landscape of the protein. All AA couples located at these positions and their dissimilarity coefficients ξ are listed in the Additional file 1: Table S1 for PR and Additional file 2: Table S2 for RT. In these tables, it appears very clearly that SDL and CM couples are not necessarily the same between treated and untreated patient sets. That is, couples can covary in one set and not in the other one (e. g. 45–46, 61–72, 63–72 for PR). Regarding the RT, the number of SL couples for the untreated set is much smaller than the number of couples in the treated set (Table 1), which means that many couples are not common to both groups. Obviously, the potential druggable targets themselves are not the same in both groups of patients (Figure 3–6). Thus, keeping this in mind, a potential drug can be able to block RT or PR in naïve patients, in treated patients or both.

How to interpret the fact that the targets we describe lie outside the active sites? The residues constituting the protein active site are generally responsible for the chemical reaction allowing the enzymatic activity of the protein. However, the active site is not the only essential part of a protein as this function is carried by its three-dimensional structure. Protease studies [33] show that its very flexible structure allows the flaps to open in order to accommodate its substrate. It is obvious that opening the flaps is an essential function for the enzymatic activity. It is therefore quite possible to block an essential function without docking a drug directly in its active site. The best examples are the existence of the non-nucleosidic reverse transcriptase inhibitors.

### In the future

We would like to develop a software able to generate a table of interdependent residues and to sort out the best AA groups to uncover inescapable drug targets. Such a strategy can be applied to any protein, especially those from RNA viruses such as flu [34], coronavirus [35], hepatitis C virus [36], provided that enough mutated sequences are available in the databases. These best interdependent AA groups could then be tested to assess whether their 3D arrangements form a druggable pocket at the protein surface. Q-SiteFinder allows a first approximation for pocket detection that will be enriched with studies that consider the flexible nature of the proteins, to discover the most suitable pockets. This technique allows the description of potential targets, which must be biologically validated, to prove they carry essential functions.

Viral fitness is one of the major aspects of the therapeutic escape along with variation and interdependence. Drugs increase the selection pressure and then alter the general mutational landscape of the target viral protein. Indeed, several positions are mutated in the treated set, which generate/maintain viral drug resistance. These new mutations can have a drastic impact on the fitness of the virus, and several other positions could also mutate to maintain/increase the fitness of these newly mutated viruses. It could be interesting to create a sequence database, where each sequence would be associated with a viral fitness measure [17], such as its average copy number in the blood. With this information in hand and based on the quasi-species theory principles [37], it would be easy to test if the existence of SDL groups in a sequence can be correlated with a low fitness (i.e. a low copy number). Thus, we could show that to escape a drug, a virus will have to make mutations within SDL groups and to pay the price for, by decreasing its replication potential.

## Conclusion

The choice of SDL and invariant positions as unique components of effective druggable targets has the ultimate aim of reducing or even eliminating drug-resistance. Our results describe two new potential targets on PR and 3 on RT. We offer an unusual strategy, since these targets are not necessarily the same for the treated and untreated patients. The drug-induced selection pressures reveal new mutations that most often, reduce the fitness of the mutated organism. Variants that possess mutations enabling them to acquire better fitness, will now be selected. These two successive waves of mutations change the general equilibrium between CM and SDL in the two patient sets, leading to different drug development strategies. In the near future, it can be important to administer different molecules to naive (never treated) patients and to treated patients.

Sometimes a single mutation allows viruses to escape treatment. If this mutation appears on a SL position, no function will be lost. That is why in the description of our target we include the invariant positions, which mutated, prevent protein function. However, if this first mutation appears alone, we reach the limit of our strategy and resistance can develop. Our target will be unusable as it will be the equivalent of the targets described in the past. However, drug docking on targets consisting of invariant residues and SL pairs, is the best way to block viral resistance.

Wet biology can only describe an existing situation where residues appear to mutate concomitantly to induce resistance against a PI. Conversely wet biology cannot assess a situation where two residues are required to mutate together to induce resistance (but entailing the loss of an essential function). Indeed, this situation never appears. Here, we have focused on the kind of couples constituted by SDL and not by CM to describe new potential protein pockets that could be bound by potential drugs. If we were able to do so, HIV virus could not escape treatment without loosing an essential function. Additional file 1: Table S1 summarizes these interdependence relationships (i.e. a look-up table describing the exact AAs forming CM or SDL).

The method described in this manuscript is applied to HIV but can be used on any sequence dataset. In fact the only limitation is the total number of mutations per position. Indeed, in order to study the ability of two positions to mutate simultaneously or not, it is necessary to prove that each of these positions is variable. RNA viruses mutate approximately 100 times faster than most other organisms. This ability allows these species to be prime candidates for our method. However, since the number of sequenced genomes being constantly increased, it is almost certain that in the near future, this method will also be used to find new drugs against bacteria for which antibiotic resistance are becoming a major problem of public health.

Most drugs have been developed based on their ability to bind active sites. They can therefore bind the active sites of similar proteins and thus generate possible side effects. Our technique allows to target regions outside of the active sites, which might help define drugs with fewer side effects.

As already said, it will be necessary to experimentally validate these bioinformatic predictions. For this, it is important to prove that the targets are essential for protein function. This question could be addressed by studying how the mutation of the residues composing the targets will affect viral activity. Small molecules binding the target at the selected positions can be found using virtual high throughput screening of large chemical libraries. Potential leads emerging from these hits may be refined by structure-activity studies. Finally, inhibition of viral activity in the presence of these molecules should validate the quality of the inhibitor.

## Methods

### Construction of sequence data sets

24656 PR sequences and 23052 RT sequences of HIV-1 subtype B, from non-treated patients were downloaded the 7^th^ of May 2013, from the Stanford University HIV drug resistance database [38] (http://hivdb.stanford.edu/). 10585 sequences, from patients treated with 1 to 9 PI were downloaded as well and 9784 RT sequences from patient with 1–7 NRTI and/or 1–4 NNRTI. The sequences of these two protein sets are full length i.e. containing the 99 positions of the PR, 560 positions for the RT.

### Identification of the accessible variant positions

In order to define the accessibility of the AAs to an external ligand (i.e. a potential drug), we computed the surface accessible to the solvent, using the ASA software [26] available at RPBS [27], based on the 3D PR structure PDB ID:1HSG [28] and 3D RT structure PDB ID:1DLO [29]. All AAs having an accessibility threshold greater than 25% are considered “accessible”.

### Recoding alignment

Previous protein alignments were recoded to focus the mutated AA status relative to a reference sequence. Each AA was compared to the AA from the ancestral sequence in the same position. It is recoded in 1 if it is equivalent to the ancestral sequence, 0 otherwise and N if it is not defined. Only positions lying on the surface of the protein and variants (ie with more than 0.3% of mutated positions) have been taken into account.

### Determination of interdependent positions

A couple were defined as interdependent if 3 of the following 4 statistical tests.

1. The Fisher exact test of covariance coded in R was used to examine each variant position pairs of PR and RT. To overcome the bias caused by the large number of tests performed, the p-values were re-adjusted using a FDR method in R. After this adjustment, only p-values > 0.05 were retained. The pairs corresponding to these p-values are black on the heatmap of Figure 1 and numbered in the black area on figure 2 for RT.
2. The D’ test measures the linkage disequilibrium [39,13,40] which is the non-random association calculation of two alleles at two loci. This D’ test has been computed for all pairs of positions variants and accessible (using as input the recoding alignments according to Wang data’s [13]). The pairs corresponding to these p-values are the “non red” on the heatmap of Figure 1 and numbered in the red area on Figure 2 for RT.
3. r^2^ [41] is an index derived from the correlation index D Lewontin [39,13,40]. Using recoding alignments, this test r^2^ has been computed in Perl according to (13, 32, 33) for all pairs of positions variants and accessible. The pairs corresponding to these p-values are black on the heatmap of Figure 1 and numbered in the green area on Figure 2 for RT.
4. This last test is a ${\chi}_{ij}^2$ that takes into account the true nature of AA and not just the fact that it is mutated or not. It is thus calculated from the protein alignment (not recoded) of the method according Noirvirt [42]. In these conditions, only couples expected more than 5 times were kept. Given a p-value of 0.05 in the sense of [42], we calculated that 6% of the couples of positions that are detected using the random shuffling method are due to multiplicity (i. e. FDR) for the three sets. The pairs corresponding to these p-values are black on the heatmap of Figure 1 and numbered in the blue area on Figure 2 for RT.

### Determination of background linkage disequilibrium (BLD)

Using DNA sequences, couples of non synonymous (A-A) and couples of synonymous mutations (S-S) were determined. A D’ coefficient were then computed from these data as explained in [13,14]. A couple were determined as free from BLD if D’(A-A)/D’(S-S) > 2. To simplify D’(A-A)/D’(S-S) is written D’_AA/SS_. The pairs corresponding to these p-values are “non brown” on the heatmap of Figure 1 for PR and numbered in the brown area on Figure 2 for RT.

### Partition the interdependent pairs in CM and SDL

When a couple was determined as interdependent, one can compute a signed dissimilarity coefficient ξ which is negative when the number of expected AA couple were superior of the number of observed couples (SDL pairs), otherwise it is a compensatory pairs (CM).

Furthermore this coefficient is here conventionally signed as follows:

If *Nobs*_*A*,*i*,*B*,*j*_ ≥ *Nex*_*A*,*i*,*B*,*j*_ then *ξ*_*A*,*i*,*B*,*j*_ = + *χ*^2^_*A*,*i*,*B*,*j*_

Otherwise it is negative

If *Nobs*_*A*,*i*,*B*,*j*_ < *Nex*_*A*,*i*,*B*,*j*_ then *ξ*_*A*,*i*,*B*,*j*_ = − *χ*^2^_*A*,*i*,*B*,*j*_

Where “A” is a specific AA at position “i”, “B” is a specific AA at position “j” and *χ*^2^_*A*,*i*,*B*,*j*_ is computed as in [42].

## Reviewers’ comments

### Reviewer’s report 1

Reviewer 1: N. Greenspan, Case Western Reserve University, United States of America

#### Reviewer’s comment

Petitjean *et al.* describe an interesting strategy for minimizing mutational escape of HIV from therapeutic agents targeting either protease (PR) or reverse transcriptase (RT). Based on amino acid sequence alignments from either treated or non-treated individuals, they identified amino acids that appear to be accessible and lethal or deleterious when simultaneously mutated (synthetic lethal, SL, or synthetic deleterious, SD, residues). The authors also identify apparently invariant PR and RT amino acids that are therefore assumed to be critical for molecular function.

The central hypothesis being pursued is that drugs able to bind to such SL/SD pairs that are in sufficient proximity to one another, plus one or more amino acids identified as invariant, on the molecular surface would serve as relatively non-mutable target sites for inhibitory drugs. Success in their objective would be of obvious value in the efforts to minimize the spread of HIV and the management of infection in individuals already carrying HIV. In the present manuscript, the authors also demonstrate that exposure to treatment modifies the PR and RT mutational landscapes.

1. Given that the contents of the present manuscript have employed methods already described in a previous article (Brouillet *et al.*, Biology Direct 2010, 5:40 doi:10.1186/1745-6150-5-40), although with an expanded range of application, I would have appreciated experimental data testing the critical assumptions of the analysis. More specific concerns are delineated below.

#### Authors’ response

The reviewers’ comments of our first article enabled us to significantly change the method used. Indeed, our previous method does not solve three important points:

- Discrimination of pairs of residues functionally interdependent of those that are due to a common ancestor. To answer to reviewer 3 of the previous article, we used a ‘D’ Lewontin derivative test. This new test is used to compare the rates of synonymous and non synonymous mutations for pairs of positions.
- Statistical studies were based on a single test. Three other tests were implemented (D’, r^2^, fisher).
- The nature of the amino acids was not taken into account and only the Boolean result “mutated/non-mutated” was calculated. New statistical tests now allow to define the exact nature of AAs forming interdependent couples.

##### Applications

All findings concerning RT are new results since the first paper concerned only the PR that has served as a control in this new study.

Two tables (RT, PR) describe the major pairs of mutations and the nature of the associated amino acids for the 4 sequence sets (Additional file 1: Tables S1 and Additional file 2: Table S2). These tables are essential for drug designers, chemists and chemoinformaticians.

Regarding the biological validation of these results: this is beyond the scope of the present paper but we are currently setting up a partnership with a HIV virology laboratory that will define the adequate experimental protocol and apply it.

#### Reviewer’s comment

2. I am not confident that 100% of “invariant” residues are in fact critical for function. For example, a putatively invariant tryptophan residue at the start of the second framework region within all immunoglobulin heavy and light chain variable domains sequenced prior to the study by J. Sharon [J Immunol. 1988 Apr 15;140(8):2666–9] was found not to be critical for function. For an antibody of known antigen specificity, Sharon mutated the Trp to Ala by site-directed mutagenesis without apparent effect on antibody reactivity for antigen.

#### Authors’ response

We agree with this comment. However, although we cannot say that every invariant site is essential for protein function, the contrary seems to be a special and not very widespread situation. All invariant amino acids or group of invariance (SL) cannot all be in this case. So, most of “invariant + SL” group are supposed underlie essential functions.

#### Reviewer’s comment

3. Another assumption critical to the authors’ thesis is that drugs able to bind the sites identified as including SL/SD and invariant residues will effectively inhibit function for either PR or RT. I would not be surprised if some such drugs would exhibit disappointing levels of inhibitory activity, so that mutational escape would not be essential for the virus to continue to replicate.

#### Authors’ response

It could indeed be the case, as it has been in the past for many other inhibitors whose development does not, however, relied on the strategy described in this paper. We can not predict in advance the strength of an inhibitor. Moreover, our method describes the target and not the inhibitors themselves.

#### Reviewer’s comment

4. Experimental testing will also help to address the concern raised by reviewers of the 2010 article by Brouillet *et al.* that evolutionary history may confound the identification of SL/SD amino acid pairs.

#### Authors’ response

It is thanks to the previous reviewer proposals that we set up a new test to select pairs of residues that interact for functional reasons and not for sharing a common ancestor (see answer to question 1). However, experimental testing is beyond the scope of this paper.

#### Reviewer’s comment

5. The authors appear to assume that synonymous mutations are selectively neutral. There are precedents for synonymous mutations that affect fitness through effects on RNA structure that influence the rate of translation or through other mechanisms [e.g., see Science. 2007 Jan 26;315(5811):525–8; reviewed in Chamary *et al.* Nat Rev Genet. 2006 Feb;7(2):98–108].

#### Authors’ response

We agree with the comment of the referee. Synonymous mutations may affect RNA secondary structure, and even (indirectly) protein translation and conformation.

How does this affect our results? Our method involves counting the non-synonymous and synonymous mutations per codon pairs. If the ratio is close to 1, we conclude that these codons are not subject to selection pressure and therefore the interdependence of residues comes from a shared common ancestor. False negatives could be obtained (ratio = 1). This requires the number of non-synonymous mutation pairs (numerator) selected by drug pressure to be similar to the pairs of synonymous mutations. Although this is possible in principle, we believe this phenomenon is less frequent than a direct impact of a mutation on the protein sequence. However, this deserves exploration in a further study.

### Reviewer’s report 2

Reviewer 2: Csaba Pal, Biological Research Center, Hungary.

#### Reviewer’s comment

The main objective of the paper is to identify intragenic pairs of residues that show synthetic lethal interactions in HIV proteins. The authors use this information to uncover drug target sites that could potentially mitigate the evolution of resistance. The manuscript is well written and the presentation of ideas goes straight to the point. The strategy followed by the authors is, to my knowledge, innovative and a valid approach to try to overcome drug resistance during HIV therapy.

In fact, such approach, due to its target specificity and efficiency, would also be beneficial to the development of therapeutic approaches with less toxic side effects to the therapy of new-borns, infants and young children, which, together with multi-drug resistance, is an important problem to be solved in HIV therapy. The idea of creating a software tool for the identification of inescapable drug targets is very important, and could help medicinal chemists to focus their research on compounds that bind to the predicted target sites.

The authors should discuss the benefits and future perspectives of the work more deeply in the paper. For example, the possibility to apply such methodology to other target proteins should also briefly be discussed in the manuscript. The authors should also discuss in vitro/in vivo validation of the reported results, including possible limitations of such studies.

#### Authors’ response

We thank the referee for these suggestions and will accordingly change the manuscript:

- This method, which is based on SL approach and not through the development of competitive inhibitors, could enable the discovery of less toxic molecules, which are necessary to treat more vulnerable patients.
- We suggest that it would be possible to do similar studies on other RNA virus proteins.
- We also explain the biological tests that will be required to validate the method and the limitations of such techniques.

These changes will be highlighted in yellow in the text.

### Reviewer’s report 3

Reviewer 3: István Simon, Institute of Enzymology, Hungary.

#### Reviewer’s comment

This paper is a follow-up of a few papers by Anne Vanet and coworkers on synthetic lethals. I recommend its publication after some revisions. First, the authors should clarify what the novel findings of this paper are.

#### Authors’ response

(See the reply to reviewer 1 comment 1).

#### Reviewer’s comment

Also the druggable nature of a target should be checked by in silico docking, using large drug datasets and fast docking programs.

#### Authors’ response

We thought to check the druggable nature of the target with in-silico docking using large datasets. However, as far as we know, all currently available docking programs need to initialize crucial parameters before being launched. Among them, there are the initial location and orientation of each chemical in the cartesian coordinates system of the target. The optimal selection of these parameters depends both on the target and on the ligand. It means that writing the script to launch the docking is a complex task, which may not be immediately successfull. Moreover, even when working on known target-ligand complexes, it is known that, most of the time, the correct pose is not retrieved as the first one ranked by the docking software, and this correct pose is more likely to be found among the ten or the twenty best poses, if ever found. Their manual analysis (e.g. with graphical tools) is difficult even for a small number of chemicals, so the automatic analysis of the results for large datasets is a complex task, too. Thus we feel that the requested *in silico* checking cannot be done at the occasion of a minor addition of the paper. Indeed, it should be the focus of a full scientific project.

#### Reviewer’s comment

There are some minor issues. The reference of the sequence data and not only the URL should be given.

#### Authors’ response

We will provide the reference of the sequence dataset.

Tang MW, Liu TF, Shafer RW. (2012). The HIVdb system for HIV-1 genotypic resistance interpretation. *Intervirology* 2012;55(2):98–101. Epub 2012 Jan 24.

#### Reviewer’s comment

Also the reference for Pymol should be included.

#### Authors’ response

The version used in this work is MacPyMOL0.99 To our knowledge, this software has never been published.

#### Reviewer’s comment

It is reasonable that some drug, which fits to the PR-NT and RT-NT cases can not be used for PR-T and RT-T cases, but it is not clear why they are not usable the other way around.

#### Authors’ response

We agree with the referee, the opposite case is more complicated to understand. In fact, we must keep in mind that the pairs of SL and CM are embedded within a complex network of pairs that evolves when an individual node of the network changes. Thus, in the treated patient sequence sets, several mutations occur in response to the selection pressure and the drugs developed for untreated patients may no longer work for most patients. These new mutations could cause a modification of the mutational landscape and reveal new pairs of SL, of course only in the treated patient sets. New drugs based on these SL should therefore be effective on treated patients and not on untreated patients.

### Re-reviewer’s report 3

#### Reviewer 3: István Simon

#### Reviewer’s comment

I understand that the authors are not willing to make the docking calculations in the present paper, so the paper can be published without it. However if they are willing to do it in a later paper, I suggest to consult the paper: Volkamer A. *et al.* Bioinformatics 28 (15) 2074–2075; 2012 to learn how to do it without prior knowledge of the binding sites.

Also, I would like to call their attention to the notice of the current distributor of PyMOL:

Like many software programs PyMOL was not published. It does not have a scientific algorithm one can publish. Still it is necessary to cite it. You can find instructions on how to do that here: http://www.pymol.org/citing

I suggest to consider it even for this paper.

## Additional files

Additional file 1: Table S1. SDL and CM pairs in PR for untreated and treated sets. Observed/expected pairs and dissimilarity coefficient for each SDL and CM important pairs for PR. Additional file 2: Table S2. SDL and CM pairs in RT for untreated and treated sets. Observed/expected pairs and dissimilarity coefficient for each SDL and CM important pairs for RT.

## Abbreviations

HIV: Human immunodeficiency virus
AIDS AIDS: acquired immunodeficiency syndrome
SL: Synthetic lethality
SD: synthetic deleterious. CM, compensatory mutation. HCV, Hepatitis C virus
SARS: Severe Acute Respiratory Syndrome
FDR: False Discovery Rate
PR: protease
PI: Protease Inhibitor
RT: reverse transcriptase
PDB: Protein Data Bank
3D: Tridimensional
